# Mir-155 is overexpressed in systemic sclerosis fibroblasts and is required for NLRP3 inflammasome-mediated collagen synthesis during fibrosis

**DOI:** 10.1186/s13075-017-1331-z

**Published:** 2017-06-17

**Authors:** Carol M. Artlett, Sihem Sassi-Gaha, Jennifer L. Hope, Carol A. Feghali-Bostwick, Peter D. Katsikis

**Affiliations:** 1 Department of Microbiology and Immunology, Drexel University College of Medicine, 2900 Queen Lane, Philadelphia, PA 19129 USA; 2 Department of Immunology, Erasmus University Medical Center, Rotterdam, The Netherlands; 3 Division of Rheumatology & Immunology, Medical University of South Carolina, Charleston, SC USA

**Keywords:** Fibrosis, NLRP3 inflammasome, miR-155, Systemic sclerosis, IL-1

## Abstract

**Background:**

Despite the important role that microRNAs (miRNAs) play in immunity and inflammation, their involvement in systemic sclerosis (SSc) remains poorly characterized. miRNA-155 (miR-155) plays a role in pulmonary fibrosis and its expression can be induced with interleukin (IL)-1β. SSc fibroblasts have activated inflammasomes that are integrally involved in mediating the myofibroblast phenotype. In light of this, we investigated whether miR-155 played a role in SSc and if its expression was dependent on inflammasome activation.

**Methods:**

miR-155 expression was confirmed in SSc dermal and lung fibroblasts by quantitative polymerase chain reaction (PCR). Wild-type and NLRP3-deficient murine fibroblasts were utilized to explore the regulation of miR-155 during inflammasome activation. miR-155-deficient fibroblasts and retroviral transductions with a miR-155 expression or control vectors were used to understand the contribution of miR-155 in fibrosis.

**Results:**

miR-155 was significantly increased and the highest expressing miRNA in SSc lung fibroblasts. Its expression was dependent on inflammasome activation as miR-155 expression could be blocked when inflammasome signaling was inhibited. In the absence of miR-155, inflammasome-mediated collagen synthesis could not be induced but was restored when miR-155 was expressed in miR-155-deficient fibroblasts.

**Conclusions:**

miR-155 is upregulated in SSc. These results suggest that the inflammasome promotes the expression of miR-155 and that miR-155 is a critical miRNA that drives fibrosis.

## Background

Systemic sclerosis (SSc) is a chronic autoimmune disease. It is characterized by uncontrolled fibrosis that is directly related to the morbidity and mortality of the disease [1]. Fibrotic lesions in SSc have persistently activated myofibroblasts and these cells mediate the excessive deposition of collagen in the dermis and visceral organs and display vascular abnormalities [2–4]. Patients typically present with an autoantibody profile that often defines disease progression and organ involvement [5, 6]. We recently found that activation of the inflammasome orchestrates the increased collagen synthesis in SSc fibroblasts [7]. We also found that inhibition of inflammasome signaling significantly abrogated the myofibroblast phenotype [7]. Based on these findings, we concluded that inflammasome activation plays a significant role in the pathogenesis of SSc fibrosis.

MicroRNAs (miRNAs) have been shown to regulate gene expression and specific miRNAs have been reported to be involved in SSc fibrosis [8–10]; however, little is known about the regulation of specific miRNAs during fibrosis. miRNA-155 (miR-155) has been identified as having immune regulatory functions and plays a critical role in innate and adaptive immune responses [11–14]. Increased miR-155 has been associated with liver [15] and lung fibrosis [16] and an additional study has shown that the downregulation of miR-155 at wound sites abrogates fibrosis [17]. miR-155 can be induced by interleukin (IL)-1β [18] and transforming growth factor (TGF)-β1 [19]. In light of the observation that IL-1β induces miR-155 and because we found that IL-1β processing by the inflammasome is elevated in SSc fibroblasts [7], we investigated whether miR-155 was overexpressed in SSc cells and whether its expression requires activation of the inflammasome. In some of these studies, bleomycin was used to activate the inflammasome and induce IL-1 processing and secretion and we used this molecule to further explore the contribution of the inflammasome activation to miR-155 expression in fibrosis. The results of these studies demonstrate that SSc fibroblasts have increased synthesis of miR-155, and that miR-155 expression is dependent on inflammasome activation. Importantly, miR-155 is required for collagen production following inflammasome activation as cells devoid of miR-155 cannot produce collagen in a fibrotic setting. This suggests that miRNAs are involved in the pathogenesis of SSc and, in particular, miR-155 may be an essential regulator of SSc fibrosis downstream of inflammasome activation.

## Methods

### Human subjects

Primary fibroblast strains were derived from SSc lung (*n* = 9) and normal lung (*n* = 5) explants. The lung fibroblasts lines were derived from Caucasian SSc patients aged 46–52 years (eight female, one male) with nonspecific interstitial pneumonia, that is usual interstitial pneumonia with or without pulmonary arterial hypertension. Control lung fibroblast lines were established from Caucasian normal individuals (three female, two male) aged 25–76 years who had all died due to head trauma. SSc skin-derived fibroblasts (*n* = 5) were established from Caucasian SSc patients with diffuse disease aged 40–50 years (four female, one male), with Scl-70 or RNA polymerase autoantibodies. Normal dermal fibroblasts (*n* = 6) were obtained from Coriell Repositories, Camden, NJ, USA (*n* = 4) or obtained from Pittsburgh (*n* = 2) and were derived from Caucasian and one Black individual aged 16–80 years of age. All of the SSc patients fulfilled the preliminary criteria for the classification of SSc [20, 21]. All human-derived fibroblasts were tested between passages 3 and 6.

### Cell culture

Normal human primary dermal fibroblasts (*n* = 4) and SSc primary dermal fibroblasts (*n* = 5) or normal human primary lung fibroblasts (*n* = 3) and SSc primary lung fibroblasts (*n* = 3) (750,000 cells/dish) were cultured in Dulbecco’s modified Eagle’s medium (DMEM; Mediatech Inc., Manassas, VA, USA) supplemented with 10% fetal bovine serum (FBS; Mediatech) and 1% penicillin/streptomycin (Mediatech). In the additional experiments, SSc fibroblasts were exposed to 20 μM caspase-1 inhibitor (Z-YVAD(OMe)-FMK (YVAD); Enzo Life Sciences, Plymouth Meeting, PA, USA) for 48 h. RNA was isolated using the Qiagen miRNeasy kit and the culture media was reserved for hydroxyproline assays according to Artlett et al. [7].

Fibroblasts cell lines from murine skin explants were established from NLRP3-deficient mice, miR-155-deficient mice, and wild-type C57BL/6 mice as previously described [7]. All fibroblasts derived from the knockout mice were on a C57BL/6 background. miR-155-deficient mice were a kind gift from Dr. Martin Turner (Babraham Institute, UK).

### miR-155 expression

miR-155 expression was measured by quantitative real-time polymerase chain reaction (RT-PCR) normalized to SNORD44 for human fibroblasts or SNORD47 for mouse fibroblasts using primers purchased from Quanta Biosciences, Gaithersburg, MD, USA. Total miRNA was reverse transcribed with qScript miRNA cDNA reaction kit (Quanta Biosciences) and quantified with PerfectCT SYBR Green Supermix (Quanta Biosciences) according to the manufacturer’s instructions.

### Fibroblast transduction

Retroviruses were produced in the Platinum-E cell line (Cell Biolabs). The miR-155-expressing MigR1-miR-155-gfp retrovirus was provided by E. Vigorito (Babraham Institute, UK). A MigR1-control-gfp retrovirus that expressed scrambled miR-155 sequence was used as the control. miR-155-deficient cells were transduced as previously described [14] but modified for fibroblasts. Briefly, fibroblasts at 50% confluency were treated with 8 μg/ml polybrene and retrovirus, centrifuged at 2000 g for 90 min, incubated at 37°C for 4 h, and then fresh media was added. Some of the dishes received 10 μM bleomycin or 50 ng/ml IL-1 receptor antagonist (IL-1RA) at 0 and 24 h post-transduction, and were then recovered for hydroxyproline analyses. Transduction efficiency of both retroviral constructs was determined by green fluorescent protein (GFP) expression to be approximately 10% using flow cytometry.

### Western blotting

Fibroblasts (C57BL/6 and miR-155KO) were cultured as described with or without 10 μM bleomycin; 200 μg of whole cell lysate was size fractionated on an 8% SDS polyacrylamide gel and the proteins transferred to a PVDF membrane (ThermoFisher Scientific, Waltham, MA, USA). Nonspecific binding sites were blocked with 5% skim milk and then probed with rabbit-anti-mouse TGF-β1 or β-actin (Santa Cruz Biotechnologies, Santa Cruz, CA, USA) overnight at 4°C. The membrane was washed and incubated with goat-anti-rabbit-HRP (Jackson Immunoresearch, West Grove, PA, USA). The horseradish peroxidase (HRP) signal was developed with SuperSignal Chemiluminescent Substrate (ThermoFisher Scientific). Band densities were quantified using ImageQuant LAS4000. TGF-β1 bands were normalized to the β-actin levels.

### Statistical analyses

The Mann Whitney *t* test or Wilcoxon matched-pairs signed-rank test were used to analyze the data by GraphPad Prism 7. A *p* value <0.05 was considered significant.

## Results

### miRNA-155 is overexpressed in SSc dermal and lung fibroblasts

**Fig. 1:**
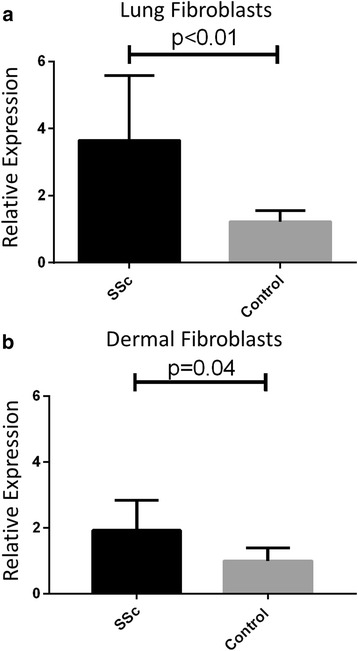
miR-155 has increased expression in systemic sclerosis (*SSc*) lung and dermal fibroblasts. **a** Lung fibroblasts (*n* = 9 SSc; *n* = 5 control) and **b** dermal fibroblasts (*n* = 5 SSc; *n* = 6 control) were assayed for miR-155 levels as described in the methods. miR-155 levels were normalized to SNORD44. Statistical analyses was by Mann-Whitney *t* test

miR-155 has been reported to be elevated in fibrosis and we wanted to determine whether this was a relevant miRNA for SSc. We found the relative expression of miR-155 in SSc lung fibroblasts (*n* = 9) to be 3.65-times more than that of normal lung fibroblasts (*n* = 5) (*p* < 0.01; Fig. 1a). We also found that SSc dermal fibroblasts (*n* = 5) had twice the relative expression of miR-155 than normal dermal fibroblasts (*n* = 6) (*p* = 0.04; Fig. 1b). These findings suggest that the increased miR-155 expression in fibroblasts may be contributing to fibrosis.

### miRNA-155 is induced by inflammasome activation

**Fig. 2:**
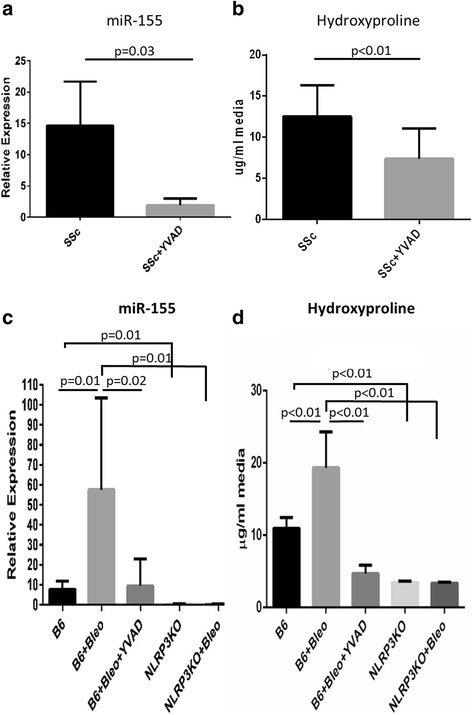
miR-155 expression requires the NLRP3 inflammasome. **a** Systemic sclerosis (*SSc*) lung fibroblasts (*n* = 7) were treated with 20 μM YVAD for 48 h. miRNA was extracted and the resulting cDNA was assayed for miR-155 levels normalized to SNORD44. **b** Hydroxyproline was measured in the culture supernatants from **a**. Statistical analyses for **a** and **b** used the Wilcoxon ranked paired *t* test. **c** In mouse cells, miR-155 expression was induced with 20 μM bleomycin (*Bleo*) + 20 μM YVAD for 48 h and analyzed for miR-155 expression normalized to SNORD47. Data are presented as the average from two independent experiments with three replicates (*n* = 6 for each condition) + SEM. **d** Hydroxyproline was measured from the culture supernatants from **c**. Statistical analyses for **c** and **d** used the Mann-Whitney *t* test

We have previously shown that SSc fibroblasts have an activated inflammasome [7] and it has been reported that miR-155 expression can be induced by IL-1β [18]. We therefore wanted to determine whether inflammasome activation could be inducing miR-155. SSc fibroblasts (*n* = 7) were cultured with the inflammasome inhibitor YVAD for 48 h. We found that in the presence of the caspase-1 inhibitor miR-155 was significantly reduced (*p* = 0.03; Fig. 2a). Further confirming our previous findings, total collagen synthesis was also significantly reduced when caspase-1 was inhibited with YVAD (*p* < 0.01; Fig. 2b). These data suggest that miR-155 expression is dependent on caspase-1 activation and that miR-155 upregulation could correlate with collagen production in SSc.

In addition, because we found the NLRP3 inflammasome to be integral in SSc fibrosis [7], we explored whether NLRP3-deficient fibroblasts (NLRP3KO) could express miR-155. B6 and NLRP3KO fibroblasts were cultured with bleomycin ± YVAD, and miR-155 expression was measured. In the wild-type B6 fibroblasts, the relative expression of miR-155 was induced with bleomycin (*p* = 0.01; Fig. 2c) and the induced expression of miR-155 could be abolished with YVAD (*p* = 0.02; Fig. 2c). Furthermore, miR-155 was not expressed in NLRP3KO fibroblasts (*p* = 0.01; Fig. 2c) and could not be induced at all with bleomycin (*p* = 0.01; Fig. 2c). Correspondingly, we then examined hydroxyproline levels and found that bleomycin induced collagen (*p* < 0.01; Fig. 2d) and YVAD blocked this process (*p* < 0.01; Fig. 2d). These data suggest that the NLRP3 inflammasome and caspase-1 may play a role in miR-155 expression in fibroblasts.

### miRNA-155 is required for inflammasome-driven collagen synthesis

**Fig. 3:**
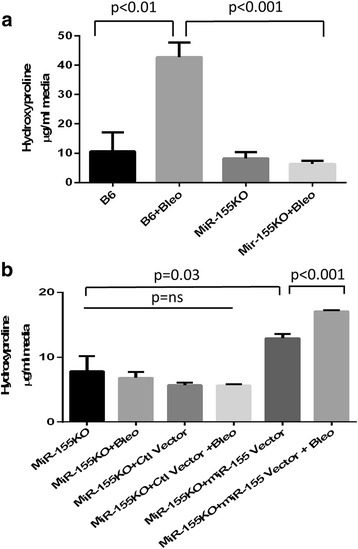
Collagen synthesis mediated by the inflammasome requires miR-155. **a** Hydroxyproline levels were measured in the culture media from B6 fibroblasts and miR-155-deficient fibroblasts (miR-155KO) + 10 μM bleomycin (*Bleo*) after 48 h. **b** Culture media hydroxyproline levels from miR-155KO fibroblasts transduced with the control (*Ctl*) vector or the miR-155 expressing vector + 10 μM Bleo after 48 h. Data for both **a** and **b** are presented as the average from two independent experiments with three replicates (*n* = 6 for each condition) + SEM using the Mann-Whitney *t* test. *ns* Not significant

Having established that both miR-155 expression and collagen production by fibroblasts were upregulated by inflammasome activation, it was important to determine whether miR-155 expression was required for collagen production during fibrosis. To address this, we first tested whether miR-155KO fibroblasts responded to bleomycin. We found that, unlike the B6 fibroblasts responding to bleomycin (*p* < 0.01; Fig. 3a), bleomycin did not induce collagen synthesis in the miR-155KO fibroblasts (*p* < 0.001; Fig. 3a).

To directly confirm this observation and to prove that miR-155 facilitates fibrosis, we transduced miR-155KO fibroblasts with a miR-155 retroviral expression vector or the retroviral control vector and then stimulated the fibroblasts with bleomycin. When cells were transduced with the control vector, collagen synthesis could not be induced with bleomycin (Fig. 3b). However, after restoration of miR-155 in the miR-155KO fibroblasts, there was a significant induction of total collagen, even without bleomycin stimulation (*p* = 0.03; Fig. 3b) and this effect was further enhanced with bleomycin (*p* < 0.001; Fig. 3b). These data indicate that miR-155 is required for fibrosis.

### miRNA-155 modulates fibrosis via IL-1 signaling

**Fig. 4:**
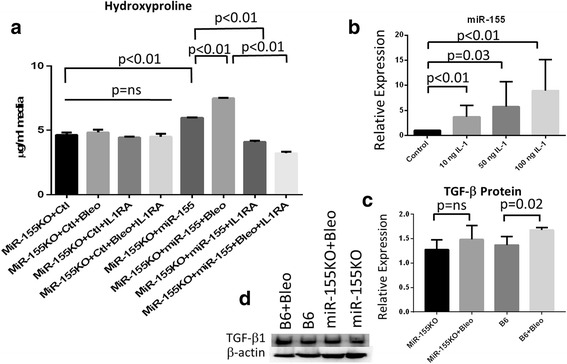
miR-155 regulates fibrosis via interleukin-1 (*IL-1*) and IL-1 induces miR-155. **a** miR-155KO fibroblasts were transduced with the miR-155 expression vector or the control (*Ctl*) vector. Some of the dishes received 10 μM bleomycin (*Bleo*) at 0 h and some of the cells also received 50 ng/ml IL-1 receptor antagonist (*IL-1RA*) at 0 and 24 h. Media was recovered after 48 h and hydroxyproline was measured (*n* = 9 replicates). **b** Dose-dependent induction of miR-155 in fibroblasts (*n* = 7 replicates). **c** Graphical representation of transforming growth factor beta (*TGF-β*) levels in miR-155KO and B6 cells ± bleo. **d** Representative Western blot (one of three samples independently tested). Data are presented as averages + SEM using the Mann-Whitney *t* test. *ns* Not significant

We found increased hydroxyproline when miR-155 was overexpressed in fibroblasts; however, only 10% of the fibroblasts were transduced by the miR-155 retrovirus (data not shown). This suggested that there might be an indirect mechanism driving the miR-155-mediated fibrotic response. IL-1 has been reported to promote miR-155 expression, and so we questioned whether miR-155 could promote IL-1 transcription that synergizes with the activation of the inflammasome; we therefore blocked the IL-1 receptor with its antagonist (IL-1RA). In repeat experiments, in the absence of miR-155, IL-1RA had no effect on hydroxyproline levels (Fig. 4a); however, in the presence of miR-155, IL-1RA completely abrogated bleomycin-induced hydroxyproline (*p* < 0.01; Fig. 4a). In addition, the spike in the level of hydroxyproline that was observed when miR-155KO fibroblasts were transduced with the miR-155 vector (without bleomycin stimulation) was also via a mechanism that entailed IL-1, as this spike was abolished with IL-1RA (*p* < 0.01; Fig. 4a).

Confirming the data by Pottier et al. [18], we found that the addition of IL-1 to fibroblasts induced miR-155 in a dose-dependent manner (Fig. 4b). However, we found this expression was not transient in fibroblasts and that miR-155 expression was still elevated at 48 h at the time point when total miRNA was isolated for the other experiments. Taken together, these data suggest that in fibroblasts activation of the inflammasome is involved in IL-1-mediated expression of miR-155 and that miR-155 synergizes with the inflammasome to drive collagen synthesis during fibrosis. Furthermore, these data also imply that miR-155 provides a feed-forward mechanism promoting IL-1 transcription that can lead to upregulated collagen synthesis via IL-1 receptor signaling and further miR-155 expression.

We also found that miR-155 was necessary for increased TGF-β1 protein levels. We stimulated C57BL/6 and the miR-155KO fibroblasts with bleomycin and measured TGF-β 1 protein levels in fibroblasts. We found that there was a significant induction of TGF-β1 in the B6 fibroblasts; however in the miR-155KO TGF-β1 could not be induced. This suggests that miR-155 is driving TGF-β1 expression and contributing to the fibrotic pathology in these cells.

## Discussion

miR-155 has been studied previously in other fibrotic conditions; however, little is known about its regulation in SSc fibrosis. Pottier et al. [18] reported that miR-155-overexpressing fibroblasts had increased motility on collagen gels suggesting that this miRNA could help to mediate wound closure, whereas the knockdown of miR-155 during wound healing abrogated fibrosis. A recently published study reports on the role of miR-155 in SSc fibrosis [22]; however, this study did not explore what caused the increase in miR-155 in SSc fibroblasts but investigated the downstream responses mediated by miR-155. Yan et al. [22] found that miR-155 regulated the Akt and Wnt/β-catenin pathways. Our study further confirms and helps to define the crucial role of miR-155 in SSc fibrosis.

miR-155 expression is upregulated by IL-1 and we explored this observation in light of our recent finding that IL-1 and the inflammasome plays a significant role in SSc fibrosis [7]. We found that miR-155 expression in SSc fibroblasts is driven by inflammasome activation since inhibition of the inflammasome signaling cascade with a caspase antagonist abolished miR-155 expression and, in turn, significantly lowered collagen (Fig. 2). Our previous study used bleomycin to activate the inflammasome and upregulate collagen via IL-1 expression [7]; therefore, in these studies, we used bleomycin to activate the inflammasome and to determine the role of the NLRP3 inflammasome in miR-155 expression. Thus, to further confirm the role of the inflammasome in miR-155 expression, we show for the first time that NLRP3KO fibroblasts cannot induce miR-155 expression when stimulated with bleomycin (Fig. 2c). Taken together, this suggests that the NLRP3 inflammasome is required for miR-155 expression. We next asked whether miR-155 participates in fibrosis and found that bleomycin cannot induce collagen synthesis in the miR-155KO fibroblasts (Fig. 3a), whereas the restoration of miR-155 using a viral vector resulted in increased collagen (Fig. 3b). IL-1 was found to induce miR-155 expression (Fig. 4b) and the synthesis of collagen in miR-155-sufficient fibroblasts, and that this was mediated via IL-1 since IL-1RA abolished these findings (Fig. 4a).

Previous research by Kong et al. [19] found that TGF-β1 upregulated the expression of miR-155, leading to altered SMAD signaling; however, another study found miR-155 to be decreased by TGF-β [18]. While the data from these findings are confounding, they suggest that the expression of miR-155 by TGF-β could be dependent on the pathological setting, e.g., fibrosis vs. wound healing, and the cells they are directly acting in. We found that, in the absence of miR-155, TGF-β was not induced, supporting the findings by Zhang et al. that miR-155 can induce TGF-β expression [23].

The data presented here imply that blockade of the IL-1 receptor or sequestration of IL-1 from the circulation could be of therapeutic benefit to SSc patients. Drugs such as kineret, rilonacept, or ilaris may prove efficacious for this, as yet, untreatable pathology. Currently, there is a placebo-controlled clinical trial underway to determine whether rilonacept could be used to treat SSc. Rilonacept sequesters IL-1 from the circulation using an antibody that binds and inactivates IL-1. Administration of IL-1RA, which blocks IL-1 from binding its receptor, has been used in various human and animal studies to prevent various organ fibroses [24–29] and it is further suggested that blockade of IL-1 signaling may also be beneficial. Furthermore, elevated IL-1 or decreased IL-1RA has been directly associated with fibrosis. Decreased expression of IL-1RA has been found in idiopathic pulmonary fibrosis and this has been specifically linked to the single nucleotide polymorphism at rs2637988 which controls expression of the gene [30, 31]. Children deficient in IL-1RA have chronic inflammation that can lead to fibrosis of the lungs or vertebra, if they survive long enough [32]. Further supporting this observation, the uncontrolled expression of IL-1 in Familial Mediterranean Fever or Muckle-Wells Syndrome has been associated with an increased risk for peritoneal fibrosis [33, 34].

## Conclusions

**Fig. 5:**
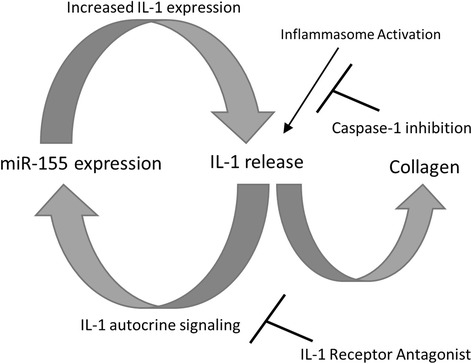
miR-155 and interleukin-1 (*IL-1*) provide a feed-forward mechanism during fibrosis. In fibroblasts, activation of the inflammasome drives miR-155 expression via IL-1 autocrine signaling that further enhances IL-1 transcription and leads to fibrosis. Blockade of the IL-1 receptor or the inflammasome abrogates this process

These data imply that miR-155 is a critical regulator in the fibrotic process and that miR-155 expression requires the NLRP3 inflammasome processing of IL-1 leading to fibrosis. Thus, we propose that the inflammasome is the initiator causing IL-1 transcription and autocrine signaling that drives the expression of miR-155 via an IL-1 signaling mechanism (Fig. 5). miR-155 synergizes with the inflammasome to induce a positive feed-forward signal that further promotes IL-1 release and autocrine signaling leading to continual collagen expression. Inhibiting either the IL-1 receptor with its antagonist or the inflammasome with YVAD breaks this cycle, and abrogates miR-155 expression and fibrosis.

## Abbreviations

B6: C57BL6
IL: Interleukin
IL-1RA: interleukin-1 receptor antagonist
miR-155: MicroRNA-155
miRNA: MicroRNA
NLRP3: NOD-like receptor family, pyrin domain containing 3
NLRP3KO: NLRP3-deficient
SSc: Systemic sclerosis
TGF: Transforming growth factor
YVAD: Z-YVAD(OMe)-FMK
